# Flavonoids furom Coreopsis tinctoria adjust lipid metabolism in hyperlipidemia animals by down-regulating adipose differentiation-related protein

**DOI:** 10.1186/1476-511X-13-193

**Published:** 2014-12-15

**Authors:** Yali Li, Xinmei Chen, Jie Xue, Jiangyun Liu, Xinhua Chen, Muhuyati Wulasihan

**Affiliations:** The Department of Internal Medicine, The First Affiliated Hospital of Xinjiang Medical University Urumqi, Xinjiang, 830000 China; The Department of Pharmacy, Shandong University of Traditional Chinese Medicine, Jinan, Shandong 250014 China; College of Pharmaceutical Sciences, Soochow University, Suzhou, Jiangsu 215123 China; The Department of Hepatobiliary and Pancreatic Surgery, The First Affiliated Hospital Zhejiang University School of Medicine, Hangzhou, Zhejiang 310003 China

**Keywords:** Coreopsis tinctoria, Flavonoid, Hyperlipemia, Fenofibrate, Adipose differentiation-related protein (ADRP)

## Abstract

**Background:**

To identify the chemical structure of Coreopsis tinctoria extracts and their effect and mechanism on reducing blood lipid in hyperlipemia mice.

**Methods:**

The flavonoids were extracted from Coreopsis tinctoria. The chemical structure was identified by HPLC. 59 mice were divided randomly into 5 groups. (group 1: normal diet control; group 2: hyperlipemia model; group 3: hyperlipemia mice treated with Coreopsis tinctoria, low dose 100 mg/kg; group 4: hyperlipemia mice treated with Coreopsis tinctoria high dose group 200 mg/kg; group 5 hyperlipemia mice treated with Fenofibrate. After 2 week of hyperlipid diet, the treatment of Coreopsis tinctoria and Fenofibrate were given for another 6 weeks with continuous hyperlipid diet. The TC, TG, HDL, histology, adipose differentiation-related protein (ADRP) expression in different groups were compared.

**Results:**

Compared with normal diet group, TC, TG in hyperlipemia model group increased ( P < 0. 01). After treatment with Coreopsis tinctoria low dose group, high dose group, TC of the hyperlipemia mice decreased (P < 0. 05) without increasing AST, ALT and ALP. Fenofibrate can also decrease TC and TG but increase AST, ALT and ALP. Expression of hepatic ADRP increased in hyperlipemia mice. Coreopsis tinctoria high dose group 200 mg/kg can inhibit ADRP as Fenofibrate does.

**Conclusion:**

The flavonoids from Coreopsis tinctoria extracts can reduce blood lipid without liver function damage, showing better anti- hyperlipemia effect than Fenofibrate by down-regulating ADRP.

## Introduction

Hyperlipidemia is clinically common disease with the rapid improvement of economy and quick changes in diet. The incidence of hyperlipidemia is threatening people’s health. A number of studies indicate that hyperlipidemia is an independent risk factor for stroke, coronary heart disease and sudden cardiac death, and is closely related to the occurrence of non-alcoholic fatty liver disease [1]. Fenofibrate helps reduce cholesterol and triglycerides in the blood to increase risk of atherosclerosis but the side effect on liver function damage are not rare [2]. Therefore, effectively and safe regulation of blood lipids and cholesterol is significant for prevention and treatment. Botanicals extract is rich of flavonoids and recent research show it plays an important role in blood lipid adjustment [3].

Coreopsis tinctoria in Xinjiang is also known as Xinjiang chrysanthemum in Snow Mountain [4]. It has multiple pharmaceutical functions such as heat-clearing, detoxifying, blood circulation promoting and blood stasis removing. Local Uyghur people use it as an herb tea to treat high blood pressure and diarrhea. Modern pharmacological studies have shown that it can adjust blood sugar, blood lipid and blood pressure [5, 6]. It is reported that Coreopsis tinctoria in Xinjiang is rich of flavonoids and can adjust glucose metabolism and reduce cardiovascular disease risk [5, 6]. There has not research on lipid metabolism [7]. In this study we investigate its active ingredient identification, biomedical function and molecular mechanism on lipid metabolism.

## Material and methods

### Instruments

Electronic scale (Mettler-Toledo manufacturing company, Model AL104). Homogeneous dispersion machine (Shanghai Jinda biochemistry Instrument CO. Model FJ-200). Spectrophotometer (Kodak, Model 722). Visible Spectrophotometer (Shanghai Scientific Instrument Company, Model 722N). High lipid diet was made of lard which was purchased commercially in local market. Cholesterol was from HuiXing, China Biochemical Reagents LLT, analytical grade, LOT number 120312. Sucrose was provided by the Yancheng Honey Garden Food CO, LOT number 20120915). Propyl thiouracil was provided by Shanghai ZhaoHui Pharmaceutical, LOT number 20120206). Propylene glycol and polysorbate-80 was provided by Reed Sinopharm Chemical Company, analytical grade, LOT number T20110418 and F20110726, respectively. Total cholesterol (TC), triglyceride (TG) and high-density lipoprotein cholesterol (HDL-c), low-density lipoprotein cholesterol (LDL-c) and alanine aminotransferase (ALT), aspartate aminotransferase (AST), alkaline phosphatase (ALP) kits are provided by Beijing Beihua Kangtai Clinical Reagent, LOT number: 20130717). Fenofibrate was used as a positive control and mixed with 0.5%CMC-Na2 to make 0.2% solution. Drugs were sealed and placed in refrigerator for the further tests.

### Process of flavonoids extract

Coreopsis tinctoria were collected from Dabanchen Xinjiang, China (LOT number 130501) and the botanical origin of material was identified by Prof. Xinmei Chen, College of Pharmaceutical Sciences, Shandong Traditional Medical University, Jinan, China. Dry flowers (20 kg) were extracted with 70% aqueous ethanol (v/v) for two times (200 L, 1.5 h each) under reflux. After evaporation, the residue was suspended in H2O (10 L) and filtrated, the liquor solution was then subjected to AB-8 macroporous resin column (80 × 10 cm i.d.), eluting with H2O, 20%, 70%, 95% ethanol (each 30 L) successively. The 70% and 95% eluants were combined and concentrated under reduced pressure to give the total flavonoid extract (PME, 114.5 g), which was applied to the following test in this study.

### HPLC analysis

The HPLC system consisted of a two solvent delivery system (LC-20AB), a vacuum degasser, a column thermostat (CTO-20A), a photodiode array detector and LC-Solution (Shimadzu Corporation, Japan). Detection wavelengths were set at 330 nm. A Cosmosil C18 AR - П column (250 mm × 4. 6 mm, 5 μm) was used with a flow rate of 1.0 mL · min-1. The injection volume was 10 μL and the column temperature was maintained at 35°C. An isocratic elution was adopted with 14% acetonitrile-0.1% acetic acid as mobile phase.

### Animal experiments for hyperlipidemia animal model

A total of 59 male mice (20 ± 2 g) were acquired from the Zhaoyan Laboratory Animal Co., Ltd. (Soochou, China). All experiments were performed in compliance with the Chinese Legislation on The Use And Care of Laboratory Animals and were approved by the committee on animal care and use of Soochow University (Laboratory animal production license: SCXK 2013–0003, laboratory animal use license: SYXK 2012–0045). Animals were maintained in Soochow University School of Medicine laboratory animal Center. Room temperature 20 ± 2°C, humidity 55-65%, 12 h/12 h light–dark cycle with free feeding and drinking water.

The non-hyperlipidemia control group were given normal diet (n = 11); hyperlipidemia anima model was induced in the mice by oral feeding of high fat diet (lard 20%, cholesterol 10%, bile salts 2%, Propylthiouracil 0.2%, propylene glycol 20%, polysorbate-80 20%.0.2 mg/per 10 g weight per day) for 2 weeks. The hyperlipidemia animal model was confirmed by blood lipid test and then the confirmed hyperlipidemia mice were randomly divided into 4 groups. With continuous high fat diet, the following treatments were administered for another 6 weeks: Hyperlipidemia group (n = 12), the low-dose group (oral feeding of Coreopsis tinctoria 100 mg/kg, n = 12), high-dose groups (oral feeding of Coreopsis tinctoria 200 mg/kg, n = 12), Fenofibrate group (oral feeding of Fenofibrate 40 mg/kg, n = 12). After the final treatment, the mice were weighed and then sacrificed. All liver specimens in each group were fixed with 10% buffered formalin and embedded in paraffin for slicing after weighing. Five sections in each group were used for histopathology examination and others for Immunohistochemical examination. Sections were stained with hematoxylin and eosin for histopathology examination. The blood sample was taken for blood lipid and liver function test.

### Blood lipid and liver function test

The serum TG, TC, LDL, HDL, ALT, AST and ALP were determined during the treatment period by serum TC and TG once every 2 weeks. After final treatment, the mice were given no food for 12 h. Blood sample was taken from orbital vein, centrifuge 15 min in 3500 r/min to get its serum for TC, TG, HDL, LDL, AST and ALT measurement. After collecting blood in mice, the liver was quickly removed and scaled for calculating index of liver (liver weight index = hepatic mass/body mass ×100%). The left lobe of the liver, was grounded at 3000 r/min in centrifuge for 10 min to get the supernatant for TC, TG measurement. The rest of hepatic tissues were snap-frozen in liquid nitrogen and stored at -80°C for Western blot.

### Morphological evaluation

Liver index was obtained by dividing liver weight by mouse weight and then times 100%. The liver specimens were fixed with 10% neutral formalin and embedded in paraffin. Paraffin sections of liver were stained with H&E for histopathological examination. Fat accumulation in liver. Was graded according to the methods (Bujanda etal., 2008; QinandTian, 2010)(grade0: no fat was found; grade1: less than 33%; grade2:33–66%; grade3:more than 66% of hepatocytes were affected by fatvacuoles).

### Western blotting analysis

Hepatic tissue samples protein concentration was determined using a BCA Kit (Pierce, Rockford, IL, USA). Samples containing 50 μg of protein each were loaded on a 10% SDS-PAGE gel and then transferred to a nitrocellulose membrane. The membrane was then rinsed and incubated with witha1:1000 dilution of a rabbit polyclonalanti mouse ADRP (Santa Cruz Biotechnology, California, USA) or anti GAPDH (1:5000 dilution) (Sigma, Missouri, USA). Membranes were then washed and incubated with horseradish-peroxidase-conjugated donkey anti-mouse IgG at 1:5000. The ECL™ Western blotting detection reagent (Amersham Biosciences, New Jersey, USA) was used for visualization and the results were analyzed quantitatively using SigmaScan Pro 5.0. The data were normalized with respect to ratios of GAPDH detected on the same blot to control for variation in protein loading across samples.

### Statistical analysis

All data are expressed as means ± SD. The significance of difference among groups was determined by using one-way ANOVA. P < 0.05 was considered statistically significant.

## Results

### Identification of flavonoids in Coreopsis tinctoria

**Figure 1:**
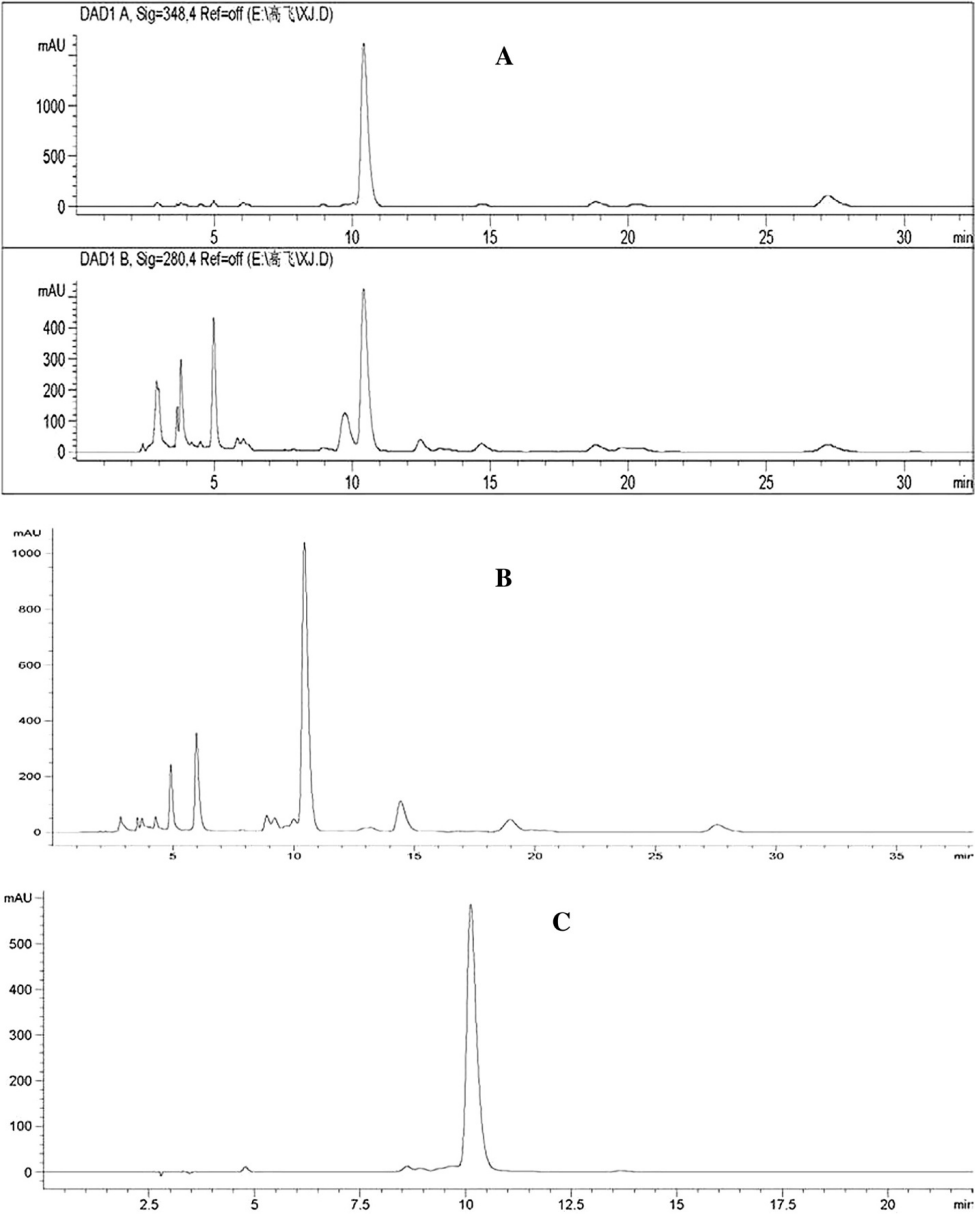
**HPLC/DAD analysis of extract from Xinjiang Coreopsis tinctoria.** HPLC chromatographic profile of Coreopsis tinctoria extracts. **(A)** Total Coreopsis tinctoria extracts from dry flower top **(B)** total flavonoid at 348 nm. **(C)** the major compound marinin at 348 nm.

Marein, 0.8653 mg/Ml was used as standard, using HPLC/PDA system to establish a qualitative and quantitative analysis of herbs and total flavonoids. Chromatographic conditions: Shimadzu LC 20A HPLC liquid systems, Cosmosil C18 column (4.6 × 250 mm), acetonitrile -0.1% ammonium acetate (20:80) as the mobile phase, flow rate 1.0 ml/min, injection volume 10 ul; PDA detection wavelength: 280,348 nm. The results shown in Figure 1. The HPLC conditions chosen were adequate to separate compounds as shown in chromatograms presented in Figure 1. The photodiode array detection (DAD) in tandem with electron spray ionization (ESI) mass spectrometry provided chemical composition of the fraction and they were mainly aglycones and glycosides of flavanone/chalcone flavonoid type. Compound marein appears as the major peak detected. The major 12 compounds seperated from LC/PDA/ESIMS chromatogram include 3,4’,5,6,7-Pentahydroxyflavanone-O-hexoside, Chlorogenic Acid, Flavanomarein, Flavanokanin, Quercetagitin-7-O-glucoside, 3,4’,5,6,7-Pentahydroxyflavanone, 3’,5,5’,7-Tetrahydroxyflavanone-O-hexoside, Marein, 3’,5,5’,7-Tetrahydroxy flavanone Okanin, Dicaffeoylquinic acid, Coreopsin.

### The changes in food intake and body weight in mice

Hyperlipidemia mice model intake less food than that in the control group (P < 0.01). Hyperlipidemia mice lost slight weight but showed no significant difference compared with control group. Throughout the experimental period, Xinjiang Coreopsis tinctoria groups showed no significant difference in food taking and body weight compared with hyperlipidemia model group, indicating that total flavonoids from Xinjiang Coreopsis tinctoria has no apparent adverse effects or acute toxicity on mice. The changes in food intake were shown in Table 1 and body weight shown in Table 2.

**Table 1:** **Average food intake changes (g/mice)**

	Normal diet (n = 11)	Hyperlipidemia (n = 12)	CT 100 mg/kg (n = 12)	CT 200 mg/kg (n = 12)	Fenofibrate (n = 12)
High lipid diet 1 week	6.19 ± 0.46**	5.36 ± 0.22	5.30 ± 0.25	5.30 ± 0.22	5.33 ± 0.23
High lipid diet 2 weeks	6.30 ± 0.89**	4.91 ± 0.58	5.12 ± 0.55	5.06 ± 0.57	4.94 ± 0.35
Treatment 1 week	6.58 ± 1.43	5.22 ± 1.16	5.56 ± 1.19	5.25 ± 1.23	5.62 ± 1.08
Treatment 2 weeks	4.62 ± 0.18	4.61 ± 0.29	4.55 ± 0.19	4.45 ± 0.24	4.57 ± 0.26
Treatment 3 weeks	5.01 ± 0.02	4.55 ± 0.47	4.56 ± 0.28	4.20 ± 0.46	4.32 ± 0.40
Treatment 4 weeks	4.77 ± 0.22	4.30 ± 0.61	4.28 ± 0.35	4.20 ± 0.49	4.20 ± 0.33
Treatment 5 weeks	4.56 ± 0.03**	3.97 ± 0.22	4.18 ± 0.19	3.99 ± 0.19	3.99 ± 0.27
Treatment 6 weeks	4.57 ± 0.02**	3.95 ± 0.16	4.12 ± 0.23	3.94 ± 0.23	4.02 ± 0.13

**P < 0.01 vs model group.

**Table 2:** **The average mouse weight change during the experiment (g/mice)**

Week	Treatment	Normal diet (n = 11)	Hyperlipidemia (n = 12)	CT 100 mg/kg (n = 12)	CT 200mg/kg (n = 12)	Fenofibrate (n = 12)
0	Begin	24.48 ± 1.80	24.03 ± 2.26	24.13 ± 2.12	24.21 ± 1.94	23.53 ± 2.06
1	High lipid diet	32.69 ± 1.91	31.96 ± 2.11	32.19 ± 1.50	31.56 ± 1.41	32.10 ± 1.88
2	High lipid diet	36.93 ± 1.81	35.43 ± 2.39	36.22 ± 2.02	35.11 ± 2.46	35.81 ± 2.75
3	treatment	40.37 ± 1.89	38.33 ± 2.89	39.06 ± 2.11	38.16 ± 2.11	37.94 ± 3.35
4	treatment	42.05 ± 2.31	39.98 ± 3.01	40.61 ± 2.45	39.45 ± 1.82	39.82 ± 3.47
5	treatment	44.11 ± 2.41	43.40 ± 3.49	43.10 ± 2.85	42.42 ± 2.22	42.03 ± 4.05
6	treatment	45.77 ± 2.39	44.33 ± 3.33	43.87 ± 2.53	43.71 ± 2.22	43.07 ± 4.35
7	treatment	47.07 ± 2.88	45.38 ± 3.47	45.66 ± 2.79	44.97 ± 2.37	44.22 ± 4.17
8	treatment	48.61 ± 3.06	46.92 ± 3.82	47.31 ± 3.02	46.83 ± 2.47	46.16 ± 4.77

### Mouse serum TG, TC, HDL, LDL change

The results are shown in Tables 3 and 4. Throughout the experimental period, TC and TG level in the hyperlipidemia mice are significantly higher than normal diet group(P < 0.05 or P < 0.01), indicating the success of the hyperlipidemia model. The total flavonoids from Coreopsis tinctoria extract decreased serum TC and TG 4 weeks after drug administration. The total flavonoids from Coreopsis tinctoria extract have no obvious impact on HDL and LDL or other adverse effects. The serum TC and TG in Fenofibrate group decreased (P < 0.05), showing no statistically significant differences in HDL levels or LDL.

**Table 3:** **The blood lipid levels at 2 weeks and 4 weeks after drug administration**

	n	TC (mmol/L)		TG (mmol/L)	
		2 week	4 week	2 week	4 week
Normal diet	11	3.4 ± 0.43**	3.28 ± 0.39**	1.56 ± 0.38**	2.19 ± 0.54*
Model	12	4.75 ± 0.69	4.53 ± 0.60	2.96 ± 0.96	2.83 ± 0.76
CT100mg/kg	12	4.39 ± 0.63	4.14 ± 0.48	2.24 ± 0.91	2.04 ± 0.68*
CT 200 mg/kg	12	4.29 ± 0.48	4.02 ± 0.38*	2.12 ± 0.55*	2.18 ± 0.68 *
Fenofibrate	12	4.05 ± 0.84*	3.70 ± 0.68**	2.72 ± 0.97	2.23 ± 0.48*

Compared with hyperlipidemia model,*P < 0.05;**P < 0.01.

**Table 4:** **The blood lipid levels at 6 weeks after drug administration**

	n	TC (mmol/L)	TG (mmol/L)	HDL (mmol/L)	LDL (mmol/L)
Normal diet	11	3.27 ± 0.34**	1.65 ± 0.27**	2.52 ± 0.21	0.05 ± 0.06*
Model	12	4.17 ± 0.70	2.69 ± 1.15	2.67 ± 0.34	0.43 ± 0.45
CT 100 mg/kg	12	3.91 ± 0.45	1.66 ± 0.55*	2.48 ± 0.19	0.67 ± 0.27
CT 200 mg/kg	12	3.88 ± 0.47	1.54 ± 0.48**	2.40 ± 0.17*	0.78 ± 0.30
Fenofibrate	12	3.62 ± 0.62*	1.92 ± 0.42*	2.51 ± 0.26	0.25 ± 0.15

Compared with hyperlipidemia control ,*P < 0.05; **P < 0.01.

### The mouse liver weight, liver weight index, liver TC and TG

The liver TC and TG in the hyperlipidemia model, the liver weight, liver weight index were significantly higher than the control group (P < 0.01) as shown in Table 5. At 6 weeks, total flavonoids from plant extract groups liver TC and liver weight index decreased (P < 0.05 or P < 0.01). Liver TG and liver weight showed no statistically significance. Fenofibrate group decreased TC (P < 0.01), but increased liver weight index and liver weight significantly (P < 0.01), no statistically significant changes in liver TG. The results are shown in Table 5.

**Table 5:** **Liver TG, Liver weight and liver weight index at 6 weeks after drug administration**

	n	TC (mg/g wet tissue)	TG (mg/g wet tissue)	Liver weight (g)	liver weight index g/100 g weight)
Normal diet	11	7.08 ± 0.48**	29.08 ± 5.73**	1.80 ± 0.16**	3.72 ± 0.31**
Model	12	16.33 ± 5.47	76.88 ± 20.04	2.46 ± 0.27	5.24 ± 0.33
CT 100 mg/kg	12	11.61 ± 1.39**	63.67 ± 9.59	2.30 ± 0.30	4.86 ± 0.46*
CT 200 mg/kg	12	12.53 ± 1.84*	66.31 ± 11.51	2.31 ± 0.22	4.96 ± 0.35*
Fenofibrate	12	11.30 ± 1.72**	77.43 ± 11.48	4.21 ± 0.64**	9.12 ± 0.93**

Compared with hyperlipidemia model,*P < 0.05;**P < 0.01.

### Effects of Coreopsis tinctoria on histopathological changes of livers

**Figure 2:**
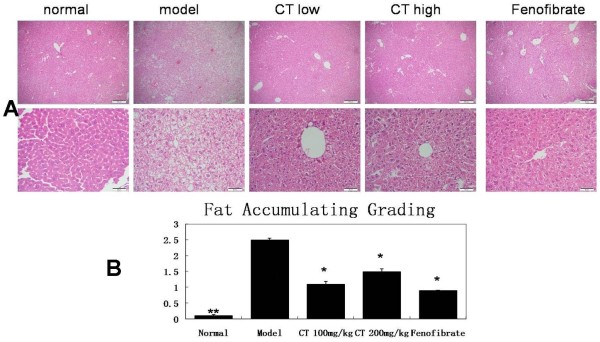
**Histopathological changes of rat liver in different groups (HE stained200X upper and 400X lower). (A)** five different treatments: Normal diet group; Model group; CT 100mg/kg group; CT 200mg/kg group; Fenofibrate group; and **(B)** Pathological grading of hepatic steatosis. Each group consisted of 11-12 mice. Values are given as means±SEM. treatment vs. normal group; *p < 0:05, **p < 0:01 vs. Model group.

**Figure 3:**
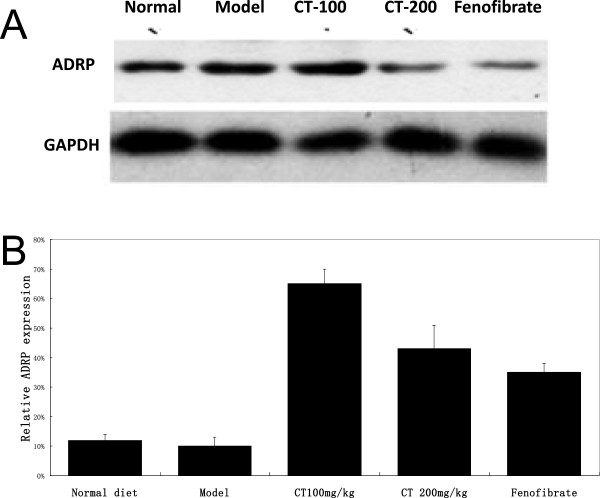
**Western blotting for ADRP expression in livers.** Representative Western blot of ADRP **(A)** as well as quantitative analysis of blots **(B)**. ADRP protein level corrected by GAPDH. Data are presented as mean _ SEM. *p < 0:05, **p < 0:01 vs. Normal group.

Photomicrographs of hepatic specimens stained with H&E were shown in Figure 2. In normal diet group, the livers showed the normal lobular structure with central veins and radiating hepatic cords, no obvious lipid deposition was found. Model fed with hyperlipid diet for 8 weeks developed a higher degree of steatosis,mostly classified as macrovesicular, with a mean grade more than 2. In CT100 mg/kg, CT200mg/kg and Fenofibrate groups, much smaller microvesicular steatosis was seen in Figure 2. The groups had much less and lower size vacuoles compared with the model group (Figure 2). Results showed that hepatic ADRP protein expressions were suppressed in flavonoid-treated groups as seen in Figure 3.

ALT and AST are the marker of liver function. In the hyperlipidemia mice group, ALT, AST and ALP showed no significant increase seen in Table 6. After given low and high dose flavonoids from Coreopsis tinctoria extract, the ALT, AST and ALP did not change significantly either. Wihile in Fenofibrate group ALT, AST, ALP increased significantly (ALT, AST, P < 0.05; ALP, P < 0.01) (seen in Table 6).

**Table 6:** **AST, ALT and ALP at 6 weeks after drug administration**

	n	ALT (u/L)	AST (u/L)	ALP (u/L)
Normal diet	11	51.88 ± 17.99	113.87 ± 6.12	101.28 ± 2.91
Model	12	53.28 ± 12.12	93.72 ± 6.44	105.27 ± 3.12
CT 100 mg/kg	12	54.12 ± 13.74	103.45 ± 7.32	102.12 ± 3.22
CT 200 mg/kg	12	61.24 ± 19.82	121 ± 8.91	104.21 ± 2.87
Fenofibrate	12	78.87 ± 18.12*	131.49 ± 6.21*	412.21 ± 21.2**

Compared with normal diet control,*P < 0.05;**P < 0.01.

## Discussion

In this study we investigate the active ingredient identification, biomedical function and molecular mechanism of Coreopsis tinctoria on lipid. An efficient method for enrichment of flavonoids from the flowers of Coreopsis tinctoria was developed using macroporous resin. HPLC proved that flavonoids are functional ingredients abundant in Coreopsis tinctoria. Flavonoids reduce TC and TG level in hyperlipidemiaadipose mice by down regulating adipose differentiation-related protein (ADRP) expression.

Flavonoids are polyphenolic compounds produced by plants and proved effective in prevention of cardiovascular, carcinogenic, neurodegenerative and immune diseases. Here we investigate its effect on modulating the lipid metabolism. Hyperlipidemia is a lipid metabolic disease due to abnormal fat metabolism [8]. In this study, we use high fat diet by intragastric administration to set up a hyperlipidemia model in mice. The serum TC and TG, LDL levels were higher than those in the control group (P < 0.05, P < 0.01), indicating successful establishment of mouse model of hyperlipidemia. Treatment with flavonoids from Coreopsis tinctoria of low and high dose groups can decrease serum TG in hyperlipidemia animal, and there is a clear dose–response relationship.

Studies have shown that lipid metabolism disorder and insulin resistance lead to liver lipid and triglycerides production increased, exceeding its transfer capacity, causing lipid accumulation in liver, or even liver damage [9]. The results of this study showed in the liver tissue of hyperlipidemia mouse model, the TC, TG, liver weight and liver weight index were higher than those in the control group. Compare with hyperlipidemia model, the low and high dose of total flavonoids can decrease the TC and liver weight indexes, indicating that total flavonoids from Coreopsis tinctoria extract can not only reduce blood fat, but also reduce the liver lipid, protecting liver function. There are chemical drugs for hyperlipidemia treatment among which Fenofibrate is a commonly used medicine. The long time use of Fenofibrate caused side effects such as liver damage and muscle breakdown [10]. In the Fenofibrate group the mice serum TC and liver tissue TG decrease, serum LDL also decreased, but it increased ALP, ALT and AST.

ALT and AST are the main index reflecting liver function status. ALT exists in the liver cells, AST exists in mitochondrial and cytosolic. Alkaline phosphatase ALP is widely distributed in many organs such as the liver, bones, intestines, kidneys and the placenta. Enzymes in the liver cells and liver sinusoids have a concentration gradient. In the case of oxidative injury, liver cell membranes were attacked by free radical and the permeability changes, leading to markedly elevated serum ALT, ALP with the involvement of mitochondria, the endoplasmic reticulum membrane system [11]. However the hyperlipidemia mice showed no ALT, AST or ALP increase, indicating the hyperlipemia is not high enough to cause changes in ALT, AST and ALP. The low and high dose groups of using total flavonoids from Coreopsis tinctoria extract, the ALT, AST and ALP do not change significantly. As a comparison, Fenofibrate group had significant ALT, AST, ALP increase, suggesting that Fenofibrate has damage in liver function. The total flavonoids of Coreopsis tinctoria extracts of liver damage has less impact on liver function.

Western-blot showed that adipose differentiation-related protein (ADRP) protein expression was higher in model model group. ADRP is expressed in the liver and its expression increases in the early stage of adipose differentiation or lipid accumulation in hepatocytes [12]. ADRP causes expansion of the number and size of lipid droplets in the liver, which results in lipid accumulation and insulin signaling inhibition. ADRP absence prevented the development of alcoholic fatty liver, associated with a reduction in the levels of triglycerides in the liver. The level of ADRP protein expression is a reliable marker for liver steatosis [12]. The present results showed that hepatic ADRP protein expressions were suppressed in flavonoid-treated groups as seen in Figure 3.

Coreopsis tinctoria Nutt.have been used traditionally in Portugal to control hyperglycaemia and a study revealed that daily administration of Coreopsis tinctoria Nutt promoted the recovery of glucose tolerance by inhibition of glucose absorption and direct promotion of insulin secretion. Dias T et al. [5] found that the bioactivity of several flavonoids in Coreopsis tinctoria extracts promote pancreatic cell function recovery through a mechanism of action other than merely antioxidant mediated. Our result confirmed that flavonoids in Coreopsis tinctoria Nutt work as bioactive metabolites with antihyperglycaemic and antihyperlipemia function.

## Conclusion

In summary, flavonoids from Coreopsis tinctoria extracts showed anti- hyperlipidemia effect, particularly in lowering triglycerides, reducing lipid deposition and protecting the liver function. The mechanism of protective effects on hepatic steatosis and liver functions is associated with regulating lipid metabolism through down-regulating blood fat via suppression ADRP expression. The experimental results provide the biomedical experiment basis for using total flavonoids from Coreopsis tinctoria extract to treat hyperlipidemia.
